# Histopathologic Patterns of Ovarian Tumors in Hawassa University Comprehensive Specialized Hospital, Southern Ethiopia

**DOI:** 10.1155/2023/2803201

**Published:** 2023-09-14

**Authors:** Tibebu Amare Abena, Fekade Yerakly, Tesfalem Korga

**Affiliations:** ^1^Wolaita Sodo University, College of Health Sciences and Medicine, Department of Pathology, Soddo, Ethiopia; ^2^Hawassa University, College of Medicine and Health Sciences, Department of Pathology, Hawassa, Ethiopia

## Abstract

**Objective:**

In Ethiopia, there is no national-level cancer registry except capital Addis Ababa, and little research was performed on ovarian tumors. This study is aimed at assessing different histopathologic patterns of ovarian tumors and their distribution based on age, biological behavior, and gross findings at a tertiary-level hospital in Ethiopia.

**Methods:**

In this study, 187 biopsy-confirmed ovarian tumors from September 2017 to August 2021 were included. All data were collected from the pathology department report format, classified according to the latest World Health Organization (WHO) classification system, and analyzed using SPSS 20.0 and Microsoft Excel 2010 at Hawassa University Comprehensive Specialized Hospital, Hawassa, Ethiopia.

**Results:**

A total of 187 women with ovarian tumors were included in this study. Of these, 143 (76.5%) were benign, 35 (18.7%) were malignant, and 9 (4.8%) were borderline tumors. Both benign and borderline tumors mostly occur at the age of 20–39 years. Surface epithelial tumors were the most common histopathologic pattern at 57.8% followed by germ cell tumors at 29.4% and sex cord-stromal tumors at 11.7%. Mature cystic teratomas were the most common benign ovarian tumors accounting for 37.8% of them, while serous cystadenocarcinomas were the most common malignant ovarian tumors accounting for 31.4% of malignant neoplasms.

**Conclusion:**

In the current study, surface epithelial tumors were the most common ovarian tumors followed by germ cell tumors. Younger age at presentation was observed for malignant ovarian tumors.

## 1. Introduction

Ovarian cancers pose a significant threat in less developed countries due to population size ranking among the top 5 cancer cases in incidence and cancer-related deaths [1]. In Ethiopian women, there were 2872 cases of ovarian cancer representing 6.2% of cancer cases, and 2313 deaths representing 7.3% of cancer deaths, ranking third next to breast and cervix cancers [2].

According to the Addis Ababa cancer registry, the age-standardized incidence rate of ovarian cancer in Ethiopia is 8.5 per 100,000 person-years which is nearly double that of other sub-Saharan countries shown by global data [1, 3].

There is no effective way of screening ovarian tumors; therefore, the majority of them present at late stages with poor survival rates making histopathologic study and staging after surgery the definitive diagnostic modality till date [4, 5]. WHO 2014 classifies them into major histopathologic diagnostic categories as epithelial tumors, sex cord-stromal tumors, germ cell tumors, mesenchymal tumors, mixed epithelial and mesenchymal tumors, and miscellaneous and secondary tumors. The distribution of these tumors histological subtypes is heterogeneous across countries [1, 6]. Data from the World Ovarian Cancer Coalition and global trends in incidence show among women having ovarian cancer; 90% of cases are surface epithelial tumors while germ cell tumors are 2-3% and sex cord-stromal tumors are 5-6%. However, in Asia and Africa, germ cell tumors account for the second most common position with 10–15% [1].

There is a distinct entity of low malignant potential ovarian tumors called borderline ovarian tumors in the subgroup of epithelial tumors. Majority of them are of serous and mucinous histology. The remaining rare subtypes are clear cell, endometroid, and brenner's bordeline tumors, accounting for < 5% of borderline ovarian tumors. Compared to overall ovarian neoplasms, borderline tumors can occur in 8.3%–23.6%. Serous borderline tumors comprise 65% of the borderline tumors while mucinous 32% [7, 8].

Even among specific groups of ovarian tumors, studies suggest that different morphological subtypes have a different pathogenesis, associated with distinct molecular alterations, different natural histories, and prognoses [9, 10]. Therefore, it is necessary that accurate pathological typing of ovarian tumors is important for prognosis and future direction of therapy. Little is known about histopathological patterns and distribution of these tumors in Africa due to a lack of adequate studies, especially in our country Ethiopia where the incidence and mortality of ovarian cancer are rising compared to our neighbors [2, 3].

## 2. Materials and Methods

### 2.1. Study Design

A descriptive cross-sectional study was conducted in HUCSH, Hawassa, Ethiopia, from September 2017 to August 2021 to study histopathologic patterns of ovarian tumors, their age distribution, gross consistency, and biologic behaviors. The study was approved after Ethical clearance, and approval was obtained from the Institutional Review Board (IRB) of Hawassa University College of Medicine and Health Science. Then, permission was obtained from the HUCSH Chief Clinical Director and the Department of Pathology to collect the required data from clients' medical record charts and report formats. Since this study is an analysis of fully anonymized data retrieved from medical records, and all necessary materials used in the process were properly returned to their original place after data collection completion, the ethics committee has waived the requirement of informed consent for both adults and minor datasets.

Clinical data and gross findings were extracted from medical records by trained histopathology technician data collectors using a structured checklist. Hematoxylin- and eosin-stained slides were retrieved and reviewed by expert pathologists. All cases of ovarian tumors were classified according to the 2014 WHO classification of ovarian tumors.

Data were checked for completeness, edited, coded, and entered into Statistical Package for Social Sciences (SPSS) version 26 by the principal investigator for further analysis. Descriptive statistics such as frequency, cross-tabulation, and chi-square were performed to investigate the morphologic patterns of ovarian tumors and to assess the presence of an association between variables. A *p* value of <0.05 was taken as the cut point to declare the presence of a statistical significance association.

### 2.2. Inclusion and Exclusion Criteria

Inclusion criteria are as follows: (1) all biopsies confirmed ovarian tumors in the HUCSH pathology department record database of specimens operated in our hospital. (2) Ovarian specimens that were received as a referral for histopathologic examination from other health facilities to our pathology department confirmed to have ovarian tumors.

Exclusion criteria are all nonneoplastic ovarian conditions, functional cysts, and cases with incomplete data records.

## 3. Results

A total of 187 ovarian tumors meeting the inclusion criteria were studied with a mean age of 35.9 years and a standard deviation of 15.33. The most affected age ranges were 20–39 years accounting for 90 (48.1%) ovarian tumors followed by 51 (27.3%) in 40–59 years, 27 (14.4%) in individuals <20 years, and 19 (10.2%) in those ≥60 years (Table 1). The majority of ovarian tumors were cystic, and more than half occurred on the right side. Most of the tumors were benign followed by malignant and borderline tumors (Table 2). Mature cystic teratoma and serous cystadenocarcinoma were the most common benign and malignant ovarian tumors, respectively (Table 3).

Considering tumor size, in this study, ovarian tumors range from the smallest 3 cm to the largest 37 cm. Only 11 (5.9%) of ovarian tumors were <5 cm in size, while the majority 90 (48.1%) were in the 10–19.9 cm size range (Table 2).

The biologic behavior of ovarian tumors and their frequency in the current study show 76.5%. 143 were benign followed by (18.7%) 35 malignant and (4.8%) 9 borderline tumors (Table 2).

Regarding tumor consistency *n* = 144, 77% are cystic in consistency followed by mixed solid and cystic 28 (15%) and solid 15 (8%). Benign tumors account for 86.8% of cystic tumors and ovarian tumors with a solid component, and the majority 58.1% were malignant (Tables 1 and 2). There is a significant association between gross consistency and behavior of ovarian tumors (*x*^2^ = 37.175, *df* = 1, *p* < 0.001).

Tumor laterality was reported in 163 cases while in 24 (12.8%) of cases, laterality was not known. Majority of cases 77% were presented unilaterally, and 10.2% were bilateral. 52.6% of bilateral ovarian tumors were malignant ovarian tumors (Tables 1 and 2).

Surface epithelial tumors were the most common histologic patterns accounting for 108 (57.8%) followed by 55 (29.4%) germ cell tumors, 22 (11.7%) sex cord-stromal tumors, and 2 (1.1%) secondary carcinomas from the gastrointestinal tract. Out of 187 ovarian tumors, mature cystic teratomas were the most common benign tumors with 54 cases and a percentage of 28.9% followed by 53 serous cystadenomas making them the second most benign tumors accounting for 28.3%. Out of 35 malignant neoplasms, the majority 21 (60%) are of surface epithelial origin followed by 11 (31.4%) malignant sex cord-stromal tumors. The remaining malignant ovarian tumors were two cases of secondary ovarian carcinomas, both from primary gastrointestinal origin, and a single case of mixed malignant germ cell tumor (Table 3).

## 4. Discussion

A total of one hundred and eighty-seven clients aged from a minimum of seven years to a maximum of 86 years, with histopathological diagnosis of ovarian tumors included in this study. The mean age at which all ovarian tumors are present is 35.9 years, and the majority 48.1% of ovarian tumors arise in the age group 20–39 years. This is similar to a study performed in Nigeria, Ethiopia, India [11–13]. Our study shows that the mean age of malignant tumors was 39.9 years, and they occurred in a wide age range with the majority 48.1% occurring >40 years, but the peak age range for them was also in 20–39 years. A study done in Nepal showed that benign tumors were occurring in the 20–40 year age range but malignant tumors occur in a higher 45–65 years age range [14]. The lower age of occurrence of malignant ovarian tumors is explained by the difference in comorbidities, risk factors, and common specific tumor types in young populations in African countries like ours [3].

The current study revealed that from a total of 187 neoplasms, 143 (76.5%) were benign, 35 (18.7%) malignant, and 9 (4.8%) borderline. This is in agreement with a study performed in India, the Kingdom of Saudi Arabia, and Ethiopia [15–17].

The present study showed that 108 (57.8%) of ovarian tumors were surface epithelial, 55 (29.4%) germ cell tumors, 22 (11.7%) sex cord-stromal tumors, and 2 (1.1%) secondary carcinomas. This is in concordance with studies performed in India and Pakistan [15, 18]. Contrary to this finding, a study in Ghana reported germ cell tumors to be the most common histologic pattern accounting for 41.9% [19].

In our study, the most common benign neoplasms were mature cystic teratomas accounting for 54 (37.8%) of benign ovarian tumors. This is similar to studies performed in Ghana and Iraq [19, 20]. In a relatively higher percentage of mature cystic teratoma, 60% noted in another study performed in Nigeria [11]. Other studies performed in western and southern India show contrasting results with serous cystadenoma being the most common benign tumor [12, 21].

In this study, serous cystadenocarcinomas were the most common malignant tumors accounting for 31.4% of malignant ovarian tumors followed by adult granulosa cell tumors at 28.6%, mucinous cystadenocarcinomas at 22.8%, endometrioid carcinoma, and secondary carcinomas each accounting 5.7%. This is in concordance with a study performed in Nigeria [22]. Another study performed in Ethiopia showed a higher percentage of serous carcinomas 52.8%, and malignant germ cell tumors were the second most common ovarian malignancy [23]. A higher number of mucinous cystadenocarcinomas was noted in the current study, and this is likely due to a lack of immunohistochemistry in our setup which could differentiate primary from secondary mucinous carcinomas [9, 24, 25].

Considering laterality, the majority 77% were unilateral tumors while 10.2% were bilateral. From unilateral tumors, the right side 43.3% was more involved than the left side 33.7% which is in concordance with studies performed in India and Bangladesh [15, 26, 27]. Information regarding laterality was not found in 24 tumors. In the current study, malignant ovarian tumors were mainly bilateral accounting for 52.6% of bilateral cases. Among malignant bilateral ovarian tumors, serous cystadenocarcinomas were the most common (45.4% bilateral) followed by 37.5% of mucinous cystadenocarcinomas, which is similar to a study conducted by Mondal et al. [28].

Tumor gross consistency wise, in our study, 144 (77%) of ovarian tumors were cystic followed by 28 (15%) mixed (solid and cystic) and 15 (8%) solid. This is in agreement with a study performed in India [29]. The present study revealed that 87.4% of benign and all borderline tumors were cystic in consistency while malignant ovarian tumors were 51.4% mixed, 28.6% cystic, and 20% solid in consistency. Similarly, other studies also concluded that malignant ovarian tumors had more solid components compared to borderline and benign tumors which had mainly cystic consistency [26, 30].

In a country lacking a proper national cancer registry and scant research activity, we believe, the current study will pave the way for more ovarian tumor studies in the future.

Despite the above-mentioned strength, immunohistochemical and molecular studies were not performed for diagnosis in the current study, though identification of TP53 mutation in high-grade serous carcinomas and demonstration of both CK7 and CK20 positivity in primary mucinous ovarian carcinomas are vital for confirmative diagnosis of these tumors [9, 10]. The cases in our study were exclusively diagnosed on the basis of morphological findings with the help of H&E stains.

The findings of this study should be interpreted in light of the inherent limitations of the study.

## 5. Conclusion

In this study, benign ovarian neoplasms are four times more prevalent compared to malignant ones. The majority of ovarian tumors are of surface epithelial origin followed by germ cell tumors and sex cord-stromal tumors. Mature cystic teratoma is the most common benign tumor while serous cystadenocarcinomas are the most common malignant ovarian tumors. The majority of ovarian tumors occur between age groups 20−39 years. We noted an early peak age of occurrence in malignant tumors and relatively a higher percentage of bilateral mucinous carcinomas.

## Figures and Tables

**Table 1:** Natural behavior of ovarian tumors by type, age, laterality, and gross consistency.

Natural behavior	Histopathologic type				Age in years				Laterality		Consistency		
	Surface epithelial	Germ cell	Sex cord	Secondary carcinoma	<20	20–39	40–59	≥60	U/L	B/L	Cystic	Mixed	Solid
Benign	78	54	11	—	23	71	39	10	117	9	125	10	8
Borderline	9	—	—	—	0	5	3	1	9	—	9	—	—
Malignant	21	1	11	2	4	14	9	8	18	10	10	18	7

**Table 2:** Natural behavior, laterality, size, and consistency of ovarian tumors.

Characteristics	Frequency	Percentage
*Natural behavior*		
Benign	143	76.5
Borderline	9	4.8
Malignant	35	18.7

*Tumor consistency*		
Cystic	144	77
Mixed	28	15
Solid	15	8

*Tumor size*		
<5 cm	11	5.9
5–9.9 cm	45	24.1
10–19.9 cm	90	48.1
≥20 cm	41	21.9

*Tumor laterality*		
Right	81	43.3
Left	63	33.7
Bilateral	19	10.2
Unknown	24	12.8

**Table 3:** Histopathologic patterns of ovarian tumors and their frequency.

Histopathologic type of tumor			Frequency (*n*)	Percentage (%)
Surface epithelial tumor	Serous 70 (64.8%)	Serous cystadenoma	53	28.3
		Serous borderline tumor	3	1.6
		Serous cystadenofibroma	3	1.6
		Serous cystadenocarcinoma	11	5.9
	Mucinous 36 (33.3%)	Mucinous cystadenoma	22	11.8
		Mucinous borderline tumor	6	3.2
		Mucinous cystadenocarcinoma	8	4.3
	Endometrioid 2 (1.9%)	Endometrioid carcinoma	2	1.1

Sex cord-stromal tumors	Granulosa cell tumor 11 (50%)	Adult granulosa cell tumor	10	5.3
		Juvenile granulosa cell tumor	1	0.5
		Fibroma	8	4.3
		Thecoma	1	0.5
		Fibrothecoma	2	1.1

Germ cell tumors		Mature cystic teratoma	54	28.9
		Mixed germ cell tumor	1	0.5

Others		Secondary carcinomas	2	1.1

## Data Availability

The data used to support the findings of this study are available from the corresponding author upon request.
